# Genetic parameters and genotype-by-environment interaction estimates for growth and feed efficiency related traits in Chinook salmon, *Oncorhynchus tshawytscha*, reared under low and moderate flow regimes

**DOI:** 10.1186/s12711-024-00929-z

**Published:** 2024-09-12

**Authors:** Leteisha A. Prescott, Megan R. Scholtens, Seumas P. Walker, Shannon M. Clarke, Ken G. Dodds, Matthew R. Miller, Jayson M. Semmens, Chris G. Carter, Jane E. Symonds

**Affiliations:** 1 Institute for Marine and Antarctic Studies, University of Tasmania, Hobart, TAS 7001 Australia; 2 Blue Economy Cooperative Research Centre, PO Box 897, Launceston, TAS 7250 Australia; 3 Cawthron Institute, Nelson, 7010 New Zealand; 4 AgResearch, Invermay Agricultural Centre, Puddle Alley, Mosgiel, 9053 New Zealand

## Abstract

**Background:**

A genotype-by-environment (G × E) interaction is defined as genotypes responding differently to different environments. In salmonids, G × E interactions can occur in different rearing conditions, including changes in salinity or temperature. However, water flow, an important variable that can influence metabolism, has yet to be considered for potential G × E interactions, although water flows differ across production stages. The salmonid industry is now manipulating flow in tanks to improve welfare and production performance, and expanding sea pen farming offshore, where flow dynamics are substantially greater. Therefore, there is a need to test whether G × E interactions occur under low and higher flow regimes to determine if industry should consider modifying their performance evaluation and selection criteria to account for different flow environments. Here, we used genotype-by-sequencing to create a genomic-relationship matrix of 37 Chinook salmon, *Oncorhynchus tshawytscha,* families to assess possible G × E interactions for production performance under two flow environments: a low flow regime (0.3 body lengths per second; bl s^−1^) and a moderate flow regime (0.8 bl s^−1^).

**Results:**

Genetic correlations for the same production performance trait between flow regimes suggest there is minimal evidence of a G × E interaction between the low and moderate flow regimes tested in this study, for Chinook salmon reared from 82.9 ± 16.8 g (${\overline{\text{x}}}$ ± s.d.) to 583.2 ± 117.1 g (${\overline{\text{x}}}$ ± s.d.). Estimates of genetic and phenotypic correlations between traits did not reveal any unfavorable trait correlations for size- (weight and condition factor) and growth-related traits, regardless of the flow regime, but did suggest measuring feed intake would be the preferred approach to improve feed efficiency because of the strong correlations between feed intake and feed efficiency, consistent with previous studies.

**Conclusion:**

This new information suggests that Chinook salmon families do not need to be selected separately for performance across different flow regimes. However, further studies are needed to confirm this across a wider range of fish sizes and flows. This information is key for breeding programs to determine if separate evaluation groups are required for different flow regimes that are used for production (e.g., hatchery, post smolt recirculating aquaculture system, or offshore).

## Background

Selective breeding programs have revolutionised production efficiency in animal farming by selecting broodstock that exhibits desirable traits [1]. Selective breeding to genetically improve production performance in salmon farming began in the 1970s [2] and since then, selecting for fast growth has achieved significant genetic gains for the industry [3–7]. Ideally, a successful breeding program generates populations that have improved performance across multiple production systems. There are several factors that can limit the success of breeding programs, one of which is environmental variation that influences genotype differences in performance. This is termed a genotype-by-environment (G × E) interaction [8]. If G × E interactions exist, genetic breeding programs can be adjusted by widening the selection criteria to include different environments to achieve improved genotype performance across multiple environments [9].

In salmon farming, determining whether G × E interactions exist is important because of the large environmental range that occurs across a production cycle. As salmonids are anadromous species (migrating from seawater to freshwater spawning grounds), commercial production begins in freshwater and ends (typically) in seawater; two environments that require opposing osmo-regulatory mechanisms [10–12]. Production stages also vary from controlled hatchery facilities (e.g., recirculating aquaculture systems (RAS) and flow-through raceways) to uncontrolled sea pens. Salinity, temperature, dissolved oxygen, and water movement are some of the abiotic factors that can vary across the entire salmonid production cycle and alter fish metabolism and activity [12–14]. Metabolism and activity can dictate growth and feed efficiency, which are key traits in selective breeding criteria, and therefore could be potential mechanisms for G × E interactions to occur.

Several G × E interactions have been identified for salmonids and other finfish species. These include performance interactions between freshwater and seawater [15–18], low and elevated temperatures [19], as well as rearing environments (e.g., pen vs. pond, tanks vs. streams, and breeding nucleus vs. test stations or commercial farms) [20–24]. Sae-Lim et al. [25] provide a review of G × E interactions in aquaculture. An environmental factor that has yet to be considered in isolation or under controlled environmental conditions but that could be linked to G × E interactions across different rearing environments, is water flow.

Water flow speeds likely vary across the salmonid production cycle. In juvenile salmon production, pre- and post-smolts can be reared in controlled tank-based RAS [26, 27] with optimal flow regimes to provide moderate exercise and improve production and animal welfare [28, 29]. In later production stages, salmonids are farmed to harvest-size in nearshore protected sites, but the industry has plans to expand into offshore high energy environments [30, 31]. This transition means that salmon will be reared in stronger environmental currents, requiring increased and sustained swimming speeds [30–33]. Investigating whether G × E interactions exist between different levels of flow is critical for the salmonid industry, as they need to determine whether flow regimes should be considered within the breeding program to improve performance across existing and future production environments.

The aims of this study were to (1) determine the phenotypic responses and genetic parameters for key performance traits when commercial Chinook salmon families are reared under two flow regimes, (2) determine if the different flow regimes result in significant G × E interactions, and (3) assess the genetic and phenotypic correlations among traits under different flow regimes. This information is important to determine how families should be evaluated, whether performance at different flows should be considered in breeding programs, and to improve genetic selection.

Two flow environments were chosen to reflect regimes that can be adopted in future RAS by the New Zealand (NZ) Chinook salmon (*Oncorhynchus tshawytscha*) industry and were based on available information, such as publications that identify flow regimes that enhance production traits in salmonids [34], previous flow regimes used with Chinook salmon as a subject species [35–38], and comparisons of swimming performance between Chinook salmon and Atlantic salmon *Salmo salar* [27, 35, 39–41].

## Methods

### Genotyping-by-sequencing

All-female pedigree Chinook salmon smolts (2020-year class) from 37 selectively bred families were sourced from Sanford’s Kaitangata commercial salmon hatchery, where the fish (age at tagging = 162 days old – 183 days old) were tagged with passive integrated transponder tags (HIDGlobal, EM4305, 12 mm long and 2 mm diameter glass tags), fin-clipped for genotyping, and transferred to the Finfish Research Centre at Cawthron Aquaculture Park, Glenduan Nelson, New Zealand on 7th December 2020. Full and half-sib families were generated from 21 sex-reversed XX sires and 32 dams from the 6th to the 25th of May 2020, with sires and dams crossed up to four and two times, respectively. The families were pooled at the eyed egg stage. A total of 3600 individually PIT tagged fish (average weight = 11.86 ± 0.04 g) were genotyped using restriction enzyme based Genotyping-by-Sequencing (GBS; PstI/MspI double digest) following the methods outlined in Dodds et al. [42], with the modifications described in Scholtens et al. [43]. TagDigger [44] was used to count the reference and alternate alleles for each variant of a previously developed catalogue of 42,839 single nucleotide polymorphisms (SNPs) [45]. Any SNPs that were monomorphic (969 SNPs) or that had no reads (11 SNPs) were removed. Six fish with mean read depth < 0.3 were also removed. Further quality control removed SNPs with minor allele frequency < 0.01, a Hardy–Weinberg (HW) disequilibrium (observed frequency of a homozygote minus its expected value) < − 0.05, or with a depth-adjusted HW test [46] *P-*value < 10^–100^. After filtering, 34,557 SNPs remained with an average call rate of 0.46 and a mean read depth of 1.31. From the 3594 genotyped fish, 3438 were successfully assigned to only one of the 37 possible families, 3191 were transferred to the finfish research facility, and 3174 fish (on average 86 offsprings per family, ranging from 44 to 113) were used in the study.

### Fish husbandry and experimental conditions

The fish were transferred into 8000 L tanks containing water with a salinity of 14 to 15 ppt at 13 ± 0.2 °C on arrival. Fish were acclimatised to full seawater (35 ppt) and a rearing temperature of 17 °C (maintained within 0.2 °C) over seventeen days. Fish were then continuously supplied with filtered recirculating seawater (35 ppt, 17 °C and maintained within 0.2 °C, and a 24 h light photoperiod). From the 29th to 31st December 2020, fish were sorted into 12 treatment tanks (8000 L) with approximately 260 fish per tank, ensuring families were evenly represented across all tanks, and tank velocities were set to 4.93 ± 0.08 cm s^−1^ for ~ 3 weeks. All fish were weighed (WT) and measured for fork length (FL) prior to the tank flow changes (average length = 174.6 ± 1.7 mm and weight = 82.90 ± 0.30 g). Tank flow regimes were then increased by 1.5 cm s^−1^ day^−1^ across three to seven days until the target speed was achieved. Tank flow regimes were maintained by directing the incoming water in a clockwise direction at either a low flow regime (LFR; 0.3 bl s^−1^) or a moderate flow regime (MFR; 0.8 bl s^−1^; six tanks per treatment). Exchange rates were maintained at 224 ± 0.07 L min^−1^ (mean ± S.E.M.). Tank flow regimes were measured daily and adjusted monthly to account for fish growth and to maintain the 0.3 bl s^−1^ and 0.8 bl s^−1^ flow regimes. Tank flow regimes were based on growth data obtained in previous experiments [47] and readjusted to match FL data during routine growth assessments.

Fish were hand fed a commercial feed (protein 37.5 g 100 g^−1^, fat 24.2 g 100 g^−1^, energy 1705 kJ 100 g^−1^) to satiation daily and pellet size was increased with fish growth, as per the manufacturer’s recommendation. Fish were fed five times per day until 1 week prior to flow regimes being set. The feeding frequency was then reduced slowly, with the fish fed three times per day for the following 2 weeks, then reduced to two feedings per day for the following 4 weeks, and then to one feeding per day for the remainder of the trial. Fish were fed once per day when feed intake rates were measured using the ballotini X-ray method described below. Tank daily feed intake (tank DFI) was measured by subtracting final feed bucket weight including uneaten pellets (retrieved by swirl separator), from the initial feed bucket weight. Uneaten pellets were counted using an automated counter (Contardor2, PFEUFFER GMBH, Kitzingen, Germany) and multiplied by the average pellet weight.

### Trait assessments

**Fig. 1:**
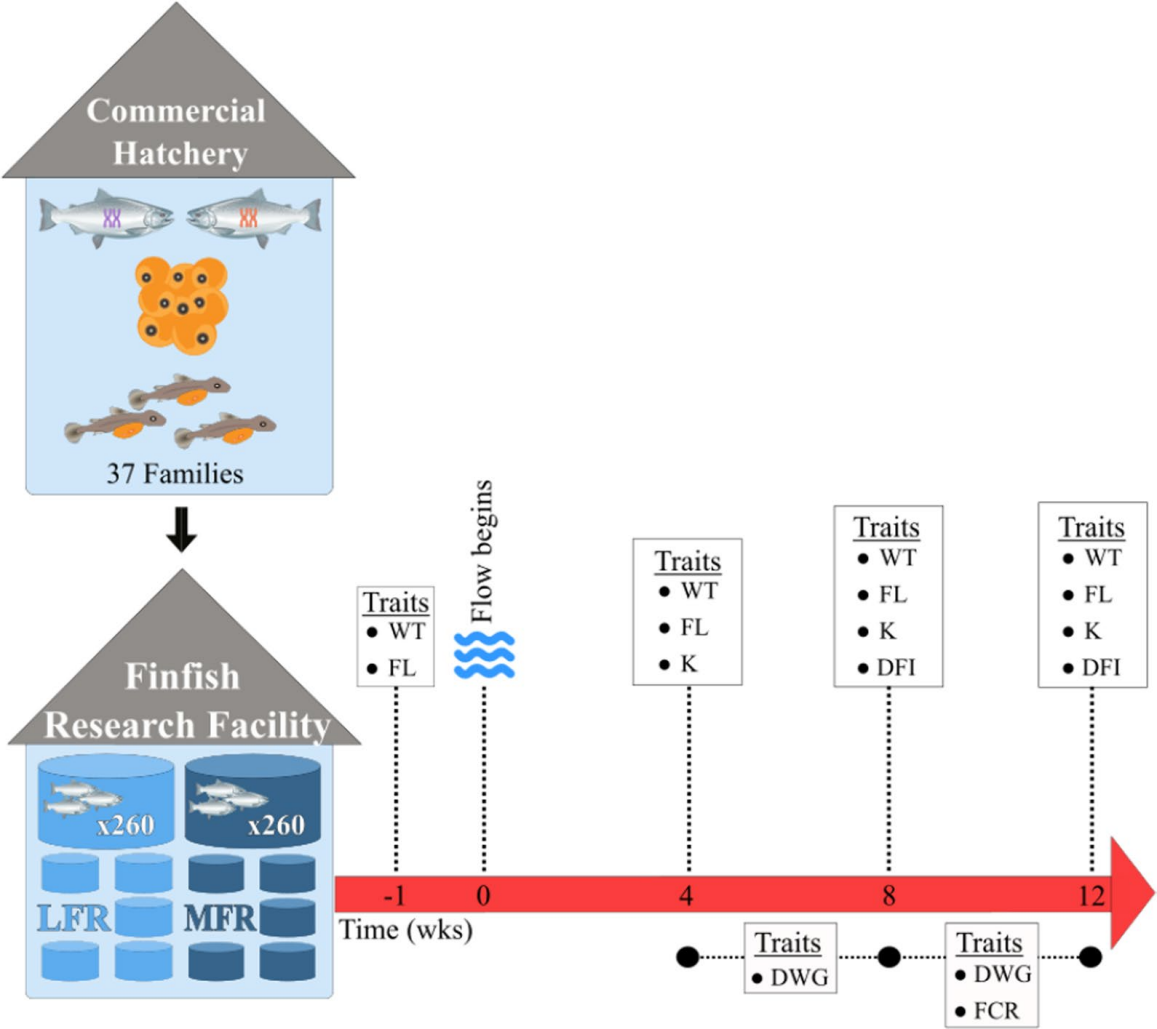
A schematic illustration depicting the experimental timeline, sampling timepoints, and the respective traits measured. Vertical dashed lines represent timing of traits measured, while horizontal dashed lines represent the period that traits were calculated across. *LFR* low flow regime, *MFR* moderate flow regime, *WT* weight, *FL* fork length, *K* condition factor, *DWG* daily weight gain, *DFI* daily feed intake, *FCR* feed conversion ratio

Figure 1 presents a schematic illustration of the sequence of sampling timepoints throughout the experiment. At 4-week intervals (4 weeks = 273–301 days old, 8 weeks = 301–329 days old, and 12 weeks = 329–357 days old), all fish were anaesthetised using tricane methanesulfonate (65 ppm; Syndel, Canada) and WT and FL of the fish were measured over two consecutive weeks (two tanks per day). Condition factors (K) and daily weight gains (DWG over two time periods: 4 to 8 and 8 to 12 weeks) were calculated as previously described in Prescott et al. [48]. A fish’s condition factor (K) was calculated as:

1 $${\text{K}} = 100000{ } \times \frac{WT}{{FL^{3} }},$$

where WT is the weight of the fish (g) and FL is the fork length (mm). Daily weight gain (DWG; g day^−1^) was calculated as:

2 $${\text{DWG}} = \frac{{WT_{f} - WT_{i} }}{days},$$

where WT_f_ is the final weight (g), WT_i_ is the initial weight (g), and days is the number of days between measurements.

At eight and 12 weeks, prior to their assessment, all fish were fed pellets (of equal composition to the feed fed daily, composition described above) containing X-ray opaque ballotini beads (~ 1 mm; fish received ballotini feed for on average 20 min 38 s, s.d. = 3 min 29 s) and were anaesthetised immediately thereafter (tricane methanesulfonate, Syndel, Canada; 65 ppm) for size measurements (as described above) and laterally radiographed [49] at 60 kV and 0.1 mAs^−1^ using an Atomscope HFX90V EX9025V portable x-ray unit (DLC Australia Pty, Ltd., Melbourne, Australia) and Canon CXDI-410C Wireless Cesium Amorphous Silicon digital radiographic receptor (DLC Australia Pty, Ltd., Melbourne, Australia; image area = 430 × 420 mm, resolution = 3408 × 3320 pixels, pixel pitch = 125 μm) set at 50 cm distance. Daily feed intake (DFI) was estimated by counting the number of beads present in the X-ray (semi-automated using “Bead Counter” software, AgResearch, NZ) and using a standard curve to convert the bead count into grams of food ingested [5, 50, 51]. Subsequently, each fish’s feed conversion ratio (FCR) was calculated following [5, 47] as:

3 $$FCR = \frac{TFI}{{MG}},$$

where *TFI* is the mean share of the meal ($\overline{SOM}$) multiplied by the total tank feed intake between the two assessments, and MG is the fish’s mass gained between the two assessments.

Share of the meal was calculated following McCarthy et al. [50] as:

4 $$SOM = \frac{DFI}{{tank DFI}}.$$

### Genetic parameters and genotype-by-environment analysis

All fish that were used in the study were included in the statistical analysis (assessment data for individuals were included unless deceased) (see Table 1 for sample size). Estimates of variance and covariance components were obtained using the Restricted Maximum Likelihood procedure in ASReml version 3 [52] fitting a univariate animal model. The model included the fixed effect of tank history and the random genetic effect of animal. Heritabilities and genomic estimated breeding values (GEBV) were obtained for each trait in each environment at each timepoint. Heritability (h^2^) was calculated as the ratio of the estimates of additive genetic variance and phenotypic variance.

**Table 1:** Descriptive statistics^1^ of production performance traits^2^ in Chinook salmon under low and moderate flow regimes

Trait	Timepoint (weeks)	Flow regime	n	${\overline{\text{X}}}$	σ	CV
WT (g)	4	Low	1583	229.8	44.3	19.3
		Moderate	1589	226.6	43.5	19.2
	8	Low	1579	397.2	76. 9	19.4
		Moderate	1574	383.9	75.46	19.6
	12	Low	1491	591.5	118.7	20.1
		Moderate	1491	575.0	115.0	20.0
FL (mm)	4	Low	1583	234.5	12.6	5.4
		Moderate	1589	233.6	12.7	5.4
	8	Low	1579	271.4	14.6	5.4
		Moderate	1574	269.5	14.6	5.4
	12	Low	1491	305.6	16.9	5.5
		Moderate	1491	303.1	16.6	5.5
K	4	Low	1583	1.8	0.1	6.6
		Moderate	1589	1.8	0.1	6.5
	8	Low	1579	2.0	0.1	7.3
		Moderate	1574	1.9	0.1	7.2
	12	Low	1491	2.1	0.2	7.8
		Moderate	1491	2.0	0.2	7.8
DWG (g)	4–8	Low	1579	167.5	36.7	21.9
		Moderate	1574	157.5	36.0	22.9
	8–12	Low	1491	194.3	48.2	24.8
		Moderate	1491	189.0	46.4	24.6
DFI (g)	8	Low	1579	8.6	3.4	39.1
		Moderate	1573	8.3	3.2	37.8
	12	Low	1491	9.6	4.3	45.3
		Moderate	1491	9.2	4.1	44.5
FCR	8–12	Low	1472	1.2	0.3	26.9
		Moderate	1474	1.2	0.3	27.3

^1^Descriptive statistics: ${\overline{\text{x}}}$, Mean; σ, standard deviation; CV, coefficient of variation

^2^Traits: WT, weight; FL, fork length; K, condition factor; DWG, daily weight gain; DFI, daily feed intake; FCR, feed conversion ratio

A bivariate model was used to estimate genetic correlations (r_g_) when treating the traits recorded in different flow regimes as separate traits, as an indicator for G × E interactions. Subsequently, because the r_g_ estimates between the two flow regimes were high, indicating they were similar genetic traits, bivariate models were fitted with a given trait being treated as the same trait in both environments (i.e., LFR and MFR). These models estimated the r_g_ and phenotypic correlations (r_p_) between traits at the same timepoint, and for the same trait at the 8- and 12-week timepoints. Rearing environment was not included in the model because it did not have a significant effect and there was minimal evidence for G × E interaction between traits. Therefore, traits (e.g., WT) measured on individuals reared under LFR and MFR were considered the same when estimating r_g_ and r_p_ between traits at the same timepoint and for the same trait at the 8- and 12-week timepoints. Age was not included in the models, as it was not found to have a significant main effect when examining all effects simultaneously.

The bivariate animal model fitted is represented as:

5 $$\left[ {\begin{array}{*{20}c} {{\mathbf{y}}_{{\text{i}}} } \\ {{\mathbf{y}}_{{\text{j}}} } \\ \end{array} } \right]{ = }\left[ {\begin{array}{*{20}c} {{\mathbf{X}}_{{\mathbf{i}}} } \\ 0 \\ \end{array} \begin{array}{*{20}c} 0 \\ {{\mathbf{X}}_{{\mathbf{j}}} } \\ \end{array} } \right]\left[ {\begin{array}{*{20}c} {{\mathbf{b}}_{{\text{i}}} } \\ {{\mathbf{b}}_{{\text{j}}} } \\ \end{array} } \right] + \left[ {\begin{array}{*{20}c} {{\mathbf{Z}}_{{\text{i}}} } \\ 0 \\ \end{array} \begin{array}{*{20}c} 0 \\ {{\mathbf{Z}}_{{\text{j}}} } \\ \end{array} } \right]\left[ {\begin{array}{*{20}c} {{\mathbf{u}}_{{\text{i}}} } \\ {{\mathbf{u}}_{{\text{j}}} } \\ \end{array} } \right] + \left[ {\begin{array}{*{20}c} {{\mathbf{e}}_{{\text{i}}} } \\ {{\mathbf{e}}_{{\text{j}}} } \\ \end{array} } \right],$$

where, for i and j, $$\left[\begin{array}{c}{\mathbf{y}}_{\text{i}}\\ {\mathbf{y}}_{\text{j}}\end{array}\right]$$ is a vector of phenotypes (for the G × E model i and j represent the different environments, i.e., LFR and MFR, and for the between trait analysis i and j represent different traits or timepoints, e.g., weight vs condition factor), $$\left[\begin{array}{c}{\mathbf{b}}_{\text{i}}\\ {\mathbf{b}}_{\text{j}}\end{array}\right]$$ is a vector for the fixed effect of the contemporary group of tank history, $$\left[\begin{array}{c}{\mathbf{u}}_{\text{i}}\\ {\mathbf{u}}_{\text{j}}\end{array}\right]$$ is a vector of random animal genetic effects, $$\left[\begin{array}{c}{\mathbf{e}}_{\text{i}}\\ {\mathbf{e}}_{\text{j}}\end{array}\right]$$ is a vector of random residuals, and **X** and **Z** are design matrices for the corresponding fixed and random effects for traits i and j. It was assumed that $$\left[ {\begin{array}{*{20}c} {{\mathbf{u}}_{{\text{i}}} } \\ {{\mathbf{u}}_{{\text{j}}} } \\ \end{array} } \right]\sim {\text{N}}\left( {\left[ {\begin{array}{*{20}c} 0 \\ 0 \\ \end{array} } \right],\left[ {\begin{array}{*{20}c} {\upsigma _{{{\text{a}}_{{\text{i}}} }}^{2} } & {\upsigma _{{{\text{a}}_{{{\text{ij}}}} }} } \\ {\upsigma _{{{\text{a}}_{{{\text{ji}}}} }} } & {\upsigma _{{{\text{a}}_{{\text{j}}} }}^{2} } \\ \end{array} } \right] \otimes {\text{G}}} \right)$$, where $$\left[\begin{array}{cc}{\upsigma }_{{\text{a}}_{\text{i}}}^{2}& {\upsigma }_{{\text{a}}_{\text{ij}}}\\ {\upsigma }_{{\text{a}}_{\text{ji}}}& {\upsigma }_{{\text{a}}_{\text{j}}}^{2}\end{array}\right]$$ is the additive genetic variance–covariance structure, and G is the genomic relationship matrix, calculated using the GBS data while taking read depth into account (following the KGD method) [42]; and $$\left[ {\begin{array}{*{20}c} {{\mathbf{e}}_{{\text{i}}} } \\ {{\mathbf{e}}_{{\text{j}}} } \\ \end{array} } \right]\sim {\text{N}}\left( {\left[ {\begin{array}{*{20}c} 0 \\ 0 \\ \end{array} } \right],\left[ {\begin{array}{*{20}c} {\upsigma _{{{\text{e}}_{{\text{i}}} }}^{2} } & {\upsigma _{{{\text{e}}_{{{\text{ij}}}} }} } \\ {\upsigma _{{{\text{e}}_{{{\text{ji}}}} }} } & {\upsigma _{{{\text{e}}_{{\text{j}}} }}^{2} } \\ \end{array} } \right] \otimes {\text{I}}} \right)$$, where $$\left[\begin{array}{cc}{\upsigma }_{{\text{e}}_{\text{i}}}^{2}& {\upsigma }_{{\text{e}}_{\text{ij}}}\\ {\upsigma }_{{\text{e}}_{\text{ji}}}& {\upsigma }_{{\text{e}}_{\text{j}}}^{2}\end{array}\right]$$ is the residual variance–covariance structure and **I** is an identity matrix.

For r_g_ estimates that were less than 0.95, a likelihood ratio test was undertaken to test whether they were significantly less than 1, with the likelihood under the null hypothesis obtained from an analysis in which r_g_ was fixed at 1. The negative of twice the difference in log likelihoods was compared to a mixture distribution consisting of half ${{\upchi }_{1}}^{2}$ and the other half having all its mass at 0 [53, 54].

## Results

### Environmental conditions and descriptive statistics

**Fig. 2:**
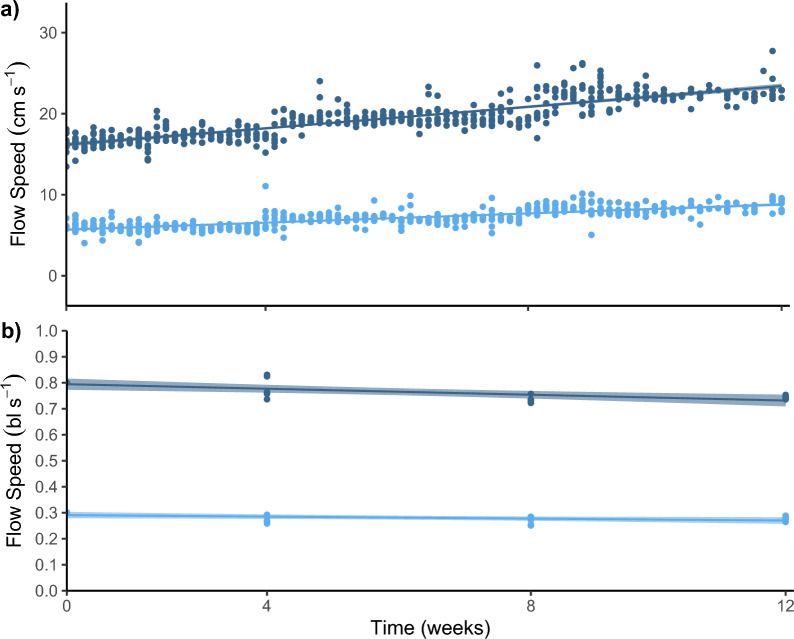
Low and moderate flow regimes during the experimental duration. Absolute (cm s^−1^) (**a**) and relative (body lengths; bl s^−1^) (**b**) flow speeds measured in Chinook salmon tank setups with low (light blue) and moderate (dark blue) flow regimes during the experimental duration (time in weeks). Tanks are represented by individual points, solid lines represent linear relationships between flow speed (in **a**) cm s^−1^, (in **b** bl s^−1^) and time (weeks), and in **b** shading represents the 99% confidence interval

The absolute flow speeds increased over time and the relative flow speed was maintained at ~ 0.8 and ~ 0.3 bl s^−1^ across the experimental duration (Fig. 2). On average, WT, K, DWG, and DFI increased over time (Table 1). The coefficient of variation was the highest for DFI and lowest for K (Table 1).

### Estimates of heritability within flow regimes

Table 2 presents estimates of the additive genetic variance, residual variance, and heritability of traits within each environment throughout the experiment and estimates of r_g_ of traits between environments at a given timepoint. Estimates of additive genetic- and residual variances for traits relating to size (i.e., WT and K) increased over time, while estimates of heritability remained similar over time and within environments. Estimates of additive genetic- and residual variances, as well as of heritability for traits relating to growth (i.e., DWG), feeding (i.e., DFI), and their ratio (i.e., FCR) also remained similar over time and within environments.

**Table 2:** Estimates of genetic parameters for traits^1^ of Chinook salmon under low and moderate flow regimes

Trait	Timepoint (weeks)	Flow regime	Additive genetic variance	Residual variance	Heritability	Genetic correlation
WT	4	Low	751.090 ± 66.456	842.478 ± 34.333	0.471 ± 0.025	0.978 ± 0.25
		Moderate	692 ± 64.896	882.819 ± 35.907	0.440 ± 0.026	
	8	Low	2556.734 ± 229.795	2876.105 ± 117.871	0.471 ± 0.025	0.995 ± 0.02
		Moderate	2297.722 ± 222.214	2970.785 ± 122.380	0.436 ± 0.027	
	12	Low	5979.066 ± 577.588	7067.323 ± 300.579	0.458 ± 0.028	0.994 ± 0.02
		Moderate	5262.617 ± 535.389	7196.552 ± 304.669	0.422 ± 0.028	
K	4	Low	0.0053 ± 0.0005	0.0065 ± 0.0003	0.452 ± 0.026	0.989 ± 0.07
		Moderate	0.0053 ± 0.0004	0.0058 ± 0.0002	0.476 ± 0.024	
	8	Low	0.0080 ± 0.0008	0.0102 ± 0.0004	0.441 ± 0.026	0.977 ± 0.02
		Moderate	0.0085 ± 0.0007	0.0088 ± 0.0004	0.491 ± 0.024	
	12	Low	0.0094 ± 0.0009	0.0122 ± 0.0005	0.435 ± 0.028	0.998 ± NA
		Moderate	0.0103 ± 0.0009	0.0113 ± 0.0005	0.476 ± 0.025	
DWG	4–8	Low	613.117 ± 58.892	750.192 ± 31.119	0.450 ± 0.027	0.995 ± 0.02
		Moderate	535.332 ± 56.770	785.792 ± 32.804	0.405 ± 0.029	
	8–12	Low	796.345 ± 98.107	1378.404 ± 60.245	0.366 ± 0.033	0.996 ± 0.02
		Moderate	750.813 ± 89.436	1337.344 ± 57.558	0.360 ± 0.031	
DFI	8	Low	1.203 ± 0.165	2.713 ± 0.115	0.307 ± 0.033	0.999 ± 0.03
		Moderate	1.066 ± 0.149	2.526 ± 0.107	0.297 ± 0.033	
	12	Low	1.346 ± 0.249	5.224 ± 0.223	0.205 ± 0.033	0.939 ± 0.06
		Moderate	0.867 ± 0.154	3.036 ± 0.131	0.222 ± 0.034	
FCR	8–12	Low	0.022 ± 0.004	0.078 ± 0.003	0.217 ± 0.033	0.967 ± 0.07
		Moderate	0.015 ± 0.003	0.082 ± 0.003	0.159 ± 0.031	

Estimates ± S.E.M

^1^Traits: WT, weight; K, condition factor; DWG, daily weight gain; DFI, daily feed intake; FCR, feed conversion ratio

*NA* Standard error was not reported due to the estimate being at its upper bound

Heritability estimates for production performance traits across each experimental timepoint were similar under LFR and MFR, with small standard errors. Heritability estimates for WT and K were the highest amongst the traits evaluated (i.e., > 0.4). For DWG and DFI, the heritability estimates were slightly lower but within a moderate to high range (i.e., 0.20 to 0.45) [55]. The heritability for FCR was estimated to be the lowest (i.e., 0.15 to 0.22) amongst the production performance traits.

### Genotype by flow regime interactions

**Fig. 3:**
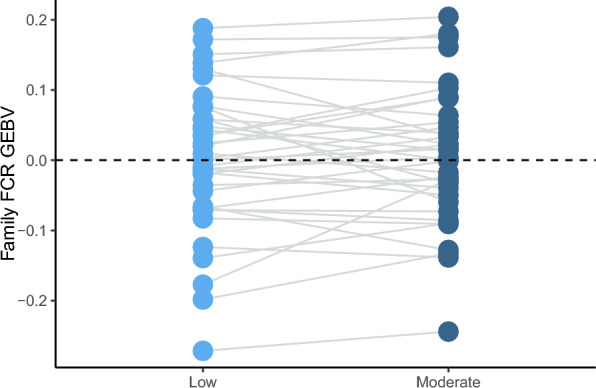
Re-ranking of family genomic estimated breeding values (GEBV) for feed conversion ratio (FCR). Light blue and dark blue points represent low and moderate flow regimes, respectively

Estimates of genetic correlations between the two flow regimes were high for most traits (> 0.85), with low standard errors (< 0.11). A re-ranking plot of family level GEBVs for FCR showed that FCR GEBVs were similar under LFR and MFR for most families, but some families appeared to re-rank across the environments, suggesting that these families may perform better or worse in different flow environments (Fig. 3).

### Genetic and phenotypic correlations between traits

Table 3 provides estimates of r_g_ and r_p_ among the traits (regardless of the flow regime) at 12 weeks. A given trait was treated as the same trait under LFR and MFR due to the high r_g_ estimates between environments presented in Table 2. Estimates of genetic and phenotypic correlations were similar at the different timepoints (results not shown), therefore only estimates at 12 weeks are reported.

**Table 3:** Estimates of genetic and phenotypic correlations for production performance traits^1^ of Chinook salmon

	WT	K	DWG	DFI	FCR
WT		0.719 ± 0.012	0.926 ± 0.003	0.468 ± 0.018	0.041 ± 0.023
K	0.661 ± 0.029		0.719 ± 0.012	0.401 ± 0.020	0.066 ± 0.023
DWG	0.947 ± 0.007	0.647 ± 0.032		0.502 ± 0.017	− 0.065 ± 0.023
DFI	0.784 ± 0.039	0.560 ± 0.051	0.811 ± 0.037		0.634 ± 0.013
FCR	0.224 ± 0.077	0.215 ± 0.073	0.126 ± 0.083	0.609 ± 0.063	

Estimates ± S.E.M for traits of Chinook salmon after 12 weeks

Genetic correlations, above diagonal; Phenotypic correlations, below diagonal

Traits under both environments were treated as the same trait

^1^ Traits: WT, weight; K, condition factor; DWG, daily weight gain; DFI, daily feed intake; FCR, feed conversion ratio

Directly measured traits, such as size (i.e., WT and K), DWG, and DFI typically showed the highest estimates of r_g_ (> 0.5). For FCR, estimates of r_g_ with other traits tended to be lower (< 0.5), with some exceptions; FCR against DFI > 0.6. Estimates of phenotypic correlations were typically lower than the respective r_g_ estimates but showed similar patterns. Size traits (i.e., WT and K) had strong correlation estimates amongst themselves and with DWG. DWG showed strong estimates of r_p_ with DFI (> 0.5), but not against FCR. DFI and FCR tended to have the lowest estimates of r_p_ with size (i.e., WT and K). FCR only present strong estimates of r_p_ with DFI.

All r_g_ estimated for traits (i.e., WT, K, DFI, and DWG) measured at week 8 and week 12 were high (0.90 to 0.98) with small standard errors (0.00 to 0.03). The estimates of r_g_ for DFI between the week 8 and week 12 timepoints was the lowest (0.90 ± 0.03), while WT was estimated to have the strongest correlation (0.98 ± 0.00).

## Discussion

The current experiment investigated whether G × E interactions exist between LFR and MFR for production performance traits in NZ-farmed Chinook salmon families. Based on high r_g_ estimates, there was minimal indication for genotype re-ranking across the two flow regimes for all traits measured. Heritability estimates were also similar for both flow environments. The re-ranking plot of family mean GEBV for FCR showed that most families had similar performance in the two environments, supporting that there is no indication of G × E interactions, although re-ranking occurred for some families. There was no evidence to suggest that families need to be selected separately for performance up to 600 g BW in different tank-based flow regimes ranging from 0.3 to 0.8 bl s^−1^.

### Genotype by flow regime interaction

Estimates of genetic correlations between the same traits under LFR and MFR were high (i.e., > 0.8) and were similar over time. A r_g_ of 0.8 or higher is often taken as an indication of a minimal G × E interactions, so then traits can be considered as the same trait in a breeding program [25, 56, 57]. However, it is important to determine whether G × E interactions are significant from both a biological and economical perspective, which can be achieved by simulating possible breeding programs and conducting cost–benefit analyses [25]. In rainbow trout, the break-even correlation has been suggested to be 0.7 [23, 25], further supporting our findings of minimal G × E interactions. Re-ranking plots for feed efficiency showed that most families had similar performance under LFR and MFR, although some families did perform differently under the two flow regimes. G × E interactions have previously been documented in other contrasting environments, for example growth and feed performance responses differ between freshwater and seawater [15–18], low and elevated temperatures [19], as well as between tank and stream environments [21, 25].

Weak re-ranking of families between LFR and MFR means that the best families in LFR were also likely to be the best families in MFR, and likewise for poorer performing families. A possible reason that flow regimes did not cause a G × E interaction in this study could be because it assessed traits during the peak growth period (< 1 kg) and before the critical size when Chinook are believed to become more sensitive to environmental factors. Another reason could be because Chinook salmon swim at similar speeds in LFR and MFR, as shown by Prescott et al. [48], and therefore, their energy expenditure was equivalent in both experiments. This study only included 37 families from one breeding program in New Zealand, and therefore, evaluating more families from multiple breeding programs is needed to confirm whether these results hold more generally.

### Genetic parameters for production performance

Heritability estimates for each trait were comparable throughout the experiment, with sampling timepoints that coincided with when post-smolt salmon undergo rapid and peak growth, from 2.4% WT day^−1^ to 1.4% WT day^−1^ (unpublished growth data on Chinook salmon in seawater) [58]. The size ranges at each sampling timepoint did overlap, which may explain the consistent heritability estimates. Scholtens et al. [45] estimated heritabilities for similar traits but in larger Chinook salmon (~ 0.9, ~ 1.5, ~ 1.9, and ~ 2.1 kg), and also showed estimates to be comparable over time. In other salmonid species (i.e., Atlantic and coho salmon *Oncorhynchus kisutch*), consistent heritabilities were also observed for production-related traits over time [59, 60].

Estimates of trait heritabilities obtained in the current experiment are comparable with those from other Chinook salmon studies [4, 5, 43, 45]. These other studies sourced families from two breeding programs that were evaluated in a range of environments from tanks to sea pens and at different times during the production cycle. In Scholtens et al. [43], estimates of r_g_ between traits in tanks versus sea pen environments ranged from 0.46 to 0.78, while heritability estimates were similar across the two environments. The combined results suggest that heritabilities are consistent throughout the production cycle and that tank-based family evaluation can be used to inform the industry’s selective breeding programs, but obtaining information from all rearing environments would be most beneficial for selection [43].

Heritability estimates for size (i.e., WT and K) and growth (i.e., DWG) traits were also comparable to those obtained for other salmonids (i.e., Atlantic and coho salmon, and rainbow trout) [17, 61] and non-salmonid fish species (i.e., Indonesian hybrid tilapia and silver trevally *Pseudocaranx georgianus*) [62, 63]. Our heritability estimates for feed related traits (i.e., DFI, SFR, and SOM) were lower [64–66] or similar to those obtained in other published fish studies [67]. Heritability estimates for FCR were similar to those obtained for Nile tilapia (*Oreochromis niloticus*) [64, 68] and sea bass (*Sparus aurata*) [69] but higher than those obtained for other salmonids (i.e., rainbow trout and European whitefish *Coregonus lavaretus* L.) [7, 65, 70]. The moderate to high heritability estimates for desired traits, such as growth, which can be easily measured in commercial settings, provides significant scope for genetic gains to be achieved through breeding programs, which can generate significant economic gains for the NZ Chinook salmon aquaculture industry.

Estimates of genetic correlations for each trait between the 8 and 12 weeks timepoints were strong, suggesting these traits remained stable through time. These results could be contributed to the measurements occurring only 4 weeks apart and during the peak growth period for NZ farmed Chinook salmon (unpublished growth data on Chinook salmon in seawater). For larger (> 985 g) NZ-farmed Chinook salmon, Scholtens et al. [45] obtained moderate estimates of r_g_ for growth rate and DFI at consecutive timepoints (i.e., time 1 compared to 2, ~ 6 to 8 weeks apart) and weaker estimates when comparing non-consecutive (i.e., time 1 compared to 3) timepoints. However, WT and K had high r_g_ estimates across all timepoints, similar to r_g_ estimated in the current experiment. In Thorland et al. [60], estimates of r_g_ for thermal growth coefficients in farmed Atlantic salmon were low across the production cycle, although estimates of r_p_ were significantly different from zero. Together, these results suggest that other factors could influence traits differently across an entire production cycle and that they may not be considered the same trait at the beginning and end of the production cycle. Farmers need to consider this when using these traits in their selection criteria, as the timing when phenotypes are measured to generate genetic parameters and for selection decisions is important.

Our estimates did not reveal unfavourable genetic correlations among the size (i.e., WT and K) and growth traits (i.e., DWG) and were comparable to estimates obtained previously for similar traits in Chinook salmon [45] and in other salmonid species [59, 71]. Harvest weight is a priority breeding objective for commercial breeding strategies in NZ [4, 45], and in our study, estimates of r_g_ and r_p_ for WT and DWG with FCR were low. In Nile tilapia the estimate of r_g_ between body weight gain and FCR was also found to be low (− 0.07) [64]. For commercial breeding programs for Chinook salmon in NZ, our results suggest that selecting families that have the largest harvest weight could lead to fish with poorer FCR. Based on the r_g_ estimated in this study, greater genetic gains for FCR would be achieved by selecting for feed intake traits rather than harvest weight or growth. However, difficulties in accurately measuring feed intake in commercial settings, along with the unfavourable r_g_ estimated in our study, makes selection for feed-efficient salmon difficult and is reflected by slow improvements for FCR in the industry [5, 45].

### Implications for future breeding programs in the context of variable flow

Stronger G × E interactions may exist in Chinook salmon if reared under different flow regimes than tested in our study, as several other aspects have yet to be determined:

1. The optimal flow regime for rearing pre- and post-smolt Chinook salmon to achieve exercise-enhanced growth has yet to be identified, where higher flow regimes may be required to achieve this [35, 38, 48]. In that case, G × E interactions may need to be re-evaluated under these flow regimes.
2. Production performance in harvest-size Chinook salmon reared under different flow regimes and/or in low and high energy farms may respond differently to that for smaller fish (as measured in our study), indicating a longer-term study with flow regimes more representative of offshore environments (e.g., faster and oscillating speeds) is needed to determine whether G × E interactions exist when farming to harvest-size.
3. Performance may also respond differently when fish are reared under different flow regimes in freshwater versus seawater (i.e., pre- versus -post smolt) and, therefore, future studies should consider G × E interactions based on both flow and salinity.

## Conclusion

Additive genetic variation is a significant component in salmon size, growth, and feed related traits, including feed intake and FCR. In the current experiment, there was minimal evidence to suggest that a G × E interaction exists for production performance between LFR and MFR for Chinook salmon with BW between 82.9 ± 0.3 and 583.2 ± 2.1 g. This demonstrates that family genetic merit is relatively consistent when individuals are reared under different flow regimes. This study provides important information for industry to consider when they integrate different tank-based environments and offshore high energy sites into their farming strategy.

## Data Availability

The dataset used and analysed during the current study are available from the corresponding author on reasonable request.
